# The EnvZ/OmpR two-component regulatory system regulates biofilm formation in *Salmonella pullorum* via interaction with the LuxS/AI-2 quorum sensing system and activation of the SoxR-AcrAB-TolC pathway

**DOI:** 10.3389/fmicb.2026.1817019

**Published:** 2026-06-01

**Authors:** Jingzhen Liang, Wenyan Chen, Can Wang, Min Wang, Ping Wei, Ziheng Xu

**Affiliations:** 1 Institute for Poultry Science and Health, Guangxi University, Nanning, China; 2 Liuzhou Center for Animal Disease Control and Prevention, Liuzhou, China; 3 Huangshan Center for Animal Disease Control and Prevention, Huangshan, China; 4 Microbiome Medicine Center, Zhujiang Hospital of Southern Medical University, Guangzhou, China; 5 School of Public Health and Management, Guangxi Key Laboratory of Translational Medicine for Treating High-incidence Infectious Diseases with Integrative Medicine, Guangxi Key Laboratory of Marine Drugs, Guangxi University of Chinese Medicine, Nanning, China

**Keywords:** AcrAB-TolC efflux pump, biofilm, EnvZ/OmpR two-component regulatory system, quorum sensing, *Salmonella pullorum*

## Abstract

To investigate the mechanisms of biofilm (BF) formation in *Salmonella pullorum* (SP), a strong BF-forming strain designated 1904D10 was selected. Differential proteomics based on tandem mass tag (TMT) labeling was performed to compare the whole bacterial proteome between planktonic and biofilm states. A total of 219 differentially expressed proteins were identified (96 up-regulated, 123 down-regulated in biofilm state). Key up-regulated proteins included those involved in the EnvZ/OmpR two-component system (TCS), LuxS/AI-2 quorum sensing (QS) system, and AcrAB-TolC efflux pump. An *ompR* deletion mutant was constructed using Red homologous recombination. Deletion of *ompR* significantly reduced biofilm formation and significantly increased susceptibility to all tested antibiotics. Electrophoretic mobility shift assay (EMSA) demonstrated that OmpR protein directly binds to the promoter region of *soxR* (a transcriptional activator of AcrAB-TolC), but not to *soxS*. Molecular docking predicted a potential interaction between the AI-2 QS signaling molecule and EnvZ protein (binding energy: −23.99 kJ/mol), with hydrogen bonds forming at five amino acid residues. This study provides evidence that OmpR directly binds to the *soxR* promoter and that AI-2 potentially interacts with EnvZ. These findings offer new mechanistic insights specific to *Salmonella pullorum*-biofilm (SP-BF) regulation and provide potential targets for biofilm control strategies.

## Graphical abstract

**Figure:**
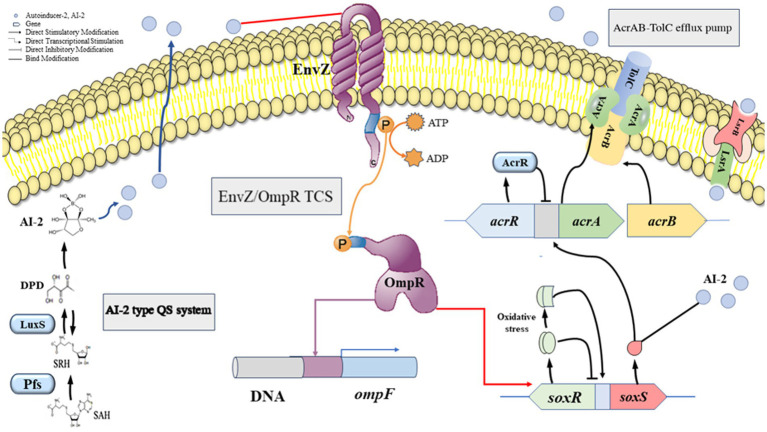

## 1 Introduction

Pullorum disease, caused by *Salmonella pullorum* (SP), is a common acute systemic infectious disease with host specificity in poultry. It remains one of the most severe diseases threatening the poultry industry globally. China is one of the world’s largest producers and consumers of broiler chickens. Guangxi Zhuang Autonomous Region boasts the richest poultry genetic resources in China. However, through years of tracking investigations, our research group has found that widespread and serious SP contamination exists among large-scale high-quality breeder chicken companies in Guangxi (Liang, 2019). In terms of SP prevention and control, aside from pullorum disease eradication programs, the primary approach is antibiotic administration. Nevertheless, SP can form biofilms (BF), which provide protection under harsh environmental conditions. Biofilms also reduce intracellular antibiotic concentrations, thereby conferring antibiotic resistance to the bacteria and increasing the difficulty of SP control. Therefore, gaining a deeper understanding of the formation mechanisms of SP biofilms is crucial for developing effective prevention and control strategies and delaying the emergence of multidrug-resistant SP (MDR-SP) strains.

Proteomics is a systemic research approach that enables a comprehensive understanding of protein expression levels, post-translational modifications, protein–protein interactions, and other aspects within living organisms. Differential proteomics can reveal physiological changes and metabolic regulatory pathways in organisms under different conditions (Pandey and Mann, 2000). Additionally, biofilm formation is regulated by systems such as the Two-Component Regulatory System (TCS), Quorum Sensing (QS), and efflux pumps. However, it remains unclear whether the EnvZ/OmpR TCS regulates SP-BF formation and whether there are inter regulatory relationships between the SP-EnvZ/OmpR TCS and other systems. Therefore, this study selects a SP strain with strong biofilm-forming ability and employs differential proteomics and AutoDock technologies to comprehensively analyze key proteins involved in SP-BF formation. The aim is to potentially uncover novel regulatory mechanisms and identify potential drug targets, thereby providing new insights and strategies for preventing SP infections.

## 2 Materials and methods

### 2.1 Source of the bacterial strain

Between 2018 and 2022, our research group collected samples from the entire production chain of six large-scale, high-quality breeder chicken companies in Guangxi. Sample processing followed the GB 4789.4–2016 national food safety standard for Salmonella detection, and the identification of Salmonella detection results was conducted using a method established by our laboratory (Wang, 2022). Over the five-year period, the research group isolated and identified a total of 627 SP strains, which are preserved in our laboratory at the Poultry Husbandry and Disease Research Institute, Guangxi University, China. From this collection, one SP isolate (1904D10) with strong biofilm-forming ability was selected for this study.

### 2.2 Determination of biofilm formation capacity of isolates

Bacterial cultures were grown in Luria-Bertani (LB) broth at 37 °C with shaking at 200 rpm unless otherwise specified. Biofilm formation capacity was assessed following the method described by Xu et al. (2016), using a 96-well microtiter plate. Each well was filled with 190 μL of Tryptic Soy Broth (TSB) and inoculated with 10 μL of bacterial suspension cultured to the logarithmic phase. Three replicate wells were set up for each isolate, along with a blank control group (uninoculated broth). The plates were incubated at 30 °C for 48 h to allow biofilm formation. After incubation, the culture medium was carefully discarded. Biofilms were gently washed three times with physiological saline to remove planktonic cells. Subsequently, 200 μL of methanol was added to each well to fix the biofilm for 10 min. Following fixation, 200 μL of 1% crystal violet solution was added, and staining was performed under light-protected conditions for 15 min. Excess stain was removed by washing with physiological saline. The bound dye was then dissolved by adding 200 μL of absolute ethanol to each well. Finally, the optical density (OD) at 595 nm (OD₅₉₅) was measured using a microplate reader to quantify biofilm formation. All optical density measurements in this study were performed at 595 nm. According to the criteria established by Stepanović et al. (2000) based on the crystal violet staining method, the mean OD value of the three replicate wells for each strain was compared with the OD value of the blank control group (cut-off value, ODc). Based on this comparison, the BF-forming capability of the strains was classified into the following four categories: strong BF-former (OD > 4 × ODc); moderate BF-former (2 × ODc < OD ≤ 4 × ODc); weak BF-former (ODc < OD ≤ 2 × ODc); and non-BF-former (OD ≤ ODc).

### 2.3 Cultivation under different conditions and sample processing of SP isolate 1904D10

The strongly biofilm-forming SP strain 1904D10, with an antimicrobial resistance profile of AMC-AMP-TMP-SIZ-SXT-NAL-STR-FFC, was selected for this study. Planktonic and biofilm-state cultures of strain 1904D10 were prepared according to the aforementioned method, each with three independent biological replicates. Differential proteomic analysis was performed in collaboration with Wuhan JinKairui Company. Following sample collection, the reaction buffer (1% SDC, 100 mM Tris–HCl pH 8.5, 10 mM TCEP, 40 mM CAA) was added to denature, reduce and alkylate the proteins in the samples. Subsequently, trypsin was used for enzymatic digestion. The digestion was then quenched, and the desalting treatment was performed. Finally, the desalted samples were lyophilized and stored at −20 °C until further use.

### 2.4 TMT-labeled differential proteomic analysis

The protein samples were labeled using TMT reagents. After labeling, the samples were pooled, desalted and dried under vacuum. The pooled sample was then fractionated by high-pH reversed-phase chromatography, and the resulting 15 fractions were combined, dried again under vacuum, and prepared for subsequent mass spectrometry analysis (Qiao, 2020). Mass spectrometry data were acquired using a Q Exactive HF-X mass spectrometer coupled with an EASY-nLC 1,200 liquid chromatography system. A 100-min gradient was established using two mobile phases: mobile phase A (0.1% formic acid) and mobile phase B (0.1% formic acid, 80% acetonitrile). Data-dependent acquisition (DDA) mode was employed, consisting of full MS scans followed by MS/MS scans (Liu, 2021). For higher-energy collisional dissociation (HCD), the normalized collision energy was set to 32%. The quadrupole isolation window was 1.2 Da, and the dynamic exclusion time was 35 s. Raw mass spectrometry data were searched against the Salmonella pullorum proteome database downloaded from UniProt (release date: 2022-09-01, containing 4,576 protein sequences) for identification and quantification. Differentially expressed proteins (DEPs) were defined as those with a fold change ≥ 1.5 and *p* < 0.05 between biofilm and planktonic states. A false discovery rate (FDR) threshold of 1% was applied to filter the results. Identified proteins were annotated with Gene Ontology (GO) terms covering biological process, cellular component, and molecular function, as well as KEGG pathways and COG categories. Annotation information was extracted for statistically differential proteins between groups, followed by functional enrichment analysis (Huang et al., 2022).

### 2.5 Construction of the *ompR* gene deletion mutant

The *ompR* gene deletion mutant of strain 1904D10 was constructed using the Red homologous recombination technique, with the primer sequences listed in Table 1. First, the plasmid pKD3 and pKD46 lyophilized powders were dissolved in deionized water and transformed into DH5α competent cells. After shaking in LB broth for 45 min, the cultures were centrifuged at 5,000 × g for 10 min at 4 °C, and the bacterial pellets were plated onto LB agar plates containing the appropriate antibiotics, followed by incubation at 37 °C for 14 h. Single colonies were selected, expanded, and plasmids were extracted according to the manufacturer’s instructions. Next, the targeting fragment was generated using pKD3 plasmid as the template. With the primer pKD3-ompR, the PCR product was obtained, followed by enzymatic digestion, gel extraction, concentration determination, and stored at −20 °C (Xu, 2022). Competent cells of 1904D10 were prepared using the CaCl₂ method. The pKD46 plasmid was chemically transformed into 1904D10 competent cells. After incubation in LB broth for 45 min, the cells were plated onto LB agar plates containing ampicillin and incubated overnight at 30 °C. PCR identification of the recombinant strain 1904D10-pKD46 was performed using primers specific for pKD46. The recombinant strain 1904D10-pKD46 was cultured at 30 °C with shaking at 180 rpm until the OD₅₉₅ reached 0.2–0.3. L-arabinose was added for induction over 1 h. Electrocompetent cells of 1904D10-pKD46 were prepared using a glycerol-based method and stored at −80 °C. The purified pKD3-ompR PCR product was electroporated into the electrocompetent 1904D10-pKD46 cells. Positive colonies were screened using primers targeting *ompR* and pKD46. Positive clones were inoculated into LB broth and incubated at 42 °C for 3 h to eliminate the pKD46 plasmid. The cultures were then streaked onto LB agar plates, and single colonies were verified by PCR, yielding the recombinant strain 1904D10-pKD3-ompR. Competent cells of this strain were prepared using glycerol, and the plasmid pCP20 was transformed into them. The *ompR* gene region was examined, and the final *ompR* deletion mutant strain 1904D10Δ*ompR* was obtained (Xu, 2022). For complementation, the *ompR* gene was amplified from the parental strain 1904D10 using primers *ompR*-C. The PCR product was digested with BamHI and SalI, ligated into an appropriate vector, and verified by sequencing. The constructed plasmid was then electroporated into the 1904D10Δ*ompR* strain (Xu, 2022).

**Table 1:** Primer sequences for red homologous recombination.

Primer name	Sequence (5′ → 3′)	Product size (bp)
*ompR*	TGCTGACCCGTGAATCTTTCCA	385
	GCTTTCAGTACCGCAAACTCCC	
pKD3-*ompR*	ATGCAAGAGAATTATAAGATTCTGGTGGTTGATGACGATATGCGTCTGCGGTGTAGGCTGGAGCTGCTT	1,016
	TATAACGCGGATGTGCCGGATCTTCTTCCACCATACGGCGCAGGCGGGAGCATATGAATATCCTCCTTAG	
pKD46	TAGCGGATCCTACCTGAC	500
	ATCAGTTCCTGTGGGTCG	
*ompR*-C	CTCGGATCCATGCAAGAGAATTATAAGATTCTGG	720
	CTCCTCGAGTCATGCTTTAGAACCGTCCGGTACG	

### 2.6 Validation of differential proteomics results

Planktonic and biofilm states of strain 1904D10 were separately cultured and processed into protein samples for validation of the differential proteomics results. For Western blot validation, 1 mL of logarithmic-phase cultures of 1904D10, 1904D10∆*ompR*, and the complementary strain CSP1904D10∆*ompR* were collected and prepared as protein lysates. The primary antibody used was a rabbit polyclonal antibody against OmpR, which was previously prepared and stored in the laboratory.

### 2.7 Biological characterization of the *ompR* gene deletion mutant

Growth curves of strains 1904D10, 1904D10ΔompR, and CSP1904D10ΔompR were determined by inoculating each strain into LB broth and incubating at 37 °C with shaking at 180 rpm. The optical density (OD) of the cultures was measured at 595 nm (OD₅₉₅) every 2 h. Biofilm formation capacity of 1904D10, 1904D10ΔompR, and CSP1904D10ΔompR was assessed using the crystal violet staining method described in section 2.2. Antimicrobial susceptibility phenotypes of the strains were evaluated by the Kirby-Bauer disk diffusion method (Xu et al., 2020).

### 2.8 Analysis of the reaction kinetics between EnvZ protein and signaling molecule AI-2

The crystal structure of the *Salmonella* EnvZ protein was retrieved from the UniProt database,^1^ followed by format processing including removal of water molecules, addition of charges and hydrogen atoms. The molecular structure of AI-2 was downloaded from the PubChem database and converted into the pdbqt format. The binding affinity between the EnvZ protein and the AI-2 molecule was then calculated using AutoDock Vina. Interaction analyses, including the identification of binding forces, were conducted. Finally, the protein–ligand interactions were visualized for further interpretation (Xu, 2022).

### 2.9 Analysis of the interaction between OmpR protein and promoters of efflux pump regulatory genes *soxS* and *soxR*

The following describes the steps of EMSA: Biotin-labeled primers were designed based on the *soxS* and *soxR* gene sequences. Probe design, synthesis, and labeling were performed by Wuhan JinKairui Biotech Company. Biotin-labeled double-stranded DNA probes were obtained by PCR amplification and annealing. Purified OmpR protein was then mixed with the probes and subjected to electrophoresis. To verify binding specificity, mutated probes (with 3-bp substitutions in the putative binding motif) and competition assays (50 × and 100 × excess of unlabeled probes) were used. The gel was transferred to a membrane using a semi-dry transfer system, followed by UV cross-linking. The membrane was blocked, incubated with HRP-conjugated streptavidin, washed, and finally developed for signal detection (Xu, 2022).

### 2.10 Statistical analysis

Gray values of Western blot bands were analyzed via ImageJ software (NIH, USA). Statistical data analysis was performed, and graphs of the results were generated with GraphPad Prism software version 8 (GraphPad Software, USA). Data are presented as mean ± standard deviation (SD) calculated from three independent biological replicates. Differences between groups were analyzed by one-way or two-way analysis of variance (ANOVA). A *p* value < 0.05 was considered statistically significant.

## 3 Results

### 3.1 Differential protein identification and quantification results between SP planktonic and BF states

**Figure 1:**
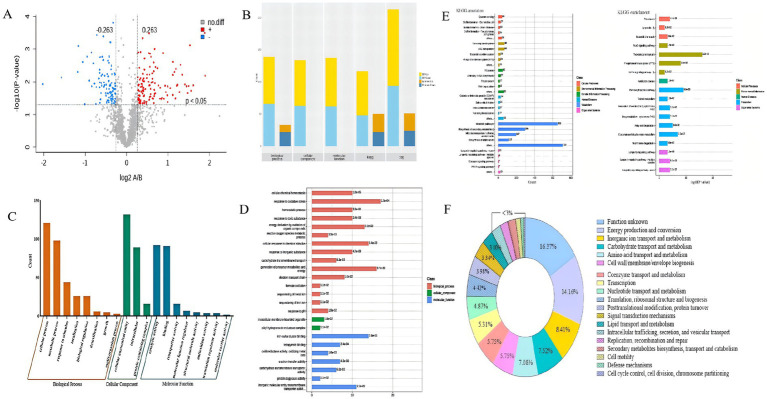
Differential Protein Identification and Quantification Results Between SP Planktonic and BF States. **(A)** The volcano plot of differentially expressed proteins in planktonic and BF states of the 1904D10 strain. **(B)** Differential protein and enrichment number statistics of strain 1904D10 in planktonic and BF states. **(C)** GO annotation of differential proteins. **(D)** GO enrichment analysis of differential proteins. **(E)** KEGG annotation and enrichment of differential proteins. (F)COG annotation of differential proteins.

For strain 1904D10 in both planktonic and biofilm states, a total of 12,483 peptides were identified, of which 12,416 were unique peptides. The number of identified proteins was 1,829, with 1,801 of them possessing quantifiable information. Differentially expressed proteins (DEPs) between the planktonic and biofilm states of strain 1904D10 were screened, yielding 219 DEPs. Among these, 96 proteins were up-regulated and 123 were down-regulated in the biofilm state compared to the planktonic state. A volcano plot depicting the DEPs between the sample groups is presented in Figure 1A. Functional analysis was performed on the screened DEPs. The number of DEPs mapped to various functional or pathway categories, as well as the count of significantly enriched proteins, are statistically summarized in Figure 1B. Table 2 lists a selection of proteins that were up-regulated in strain 1904D10 upon biofilm formation relative to its planktonic state.

**Table 2:** Partially upregulated proteins expressed in the 1904D10-BF state compared to the planktonic state.

Protein name	Protein description	Protein function	*p*-value
OmpF	Bacterial outer membrane porin	Regulates osmotic pressure	0.007
OmpR	Bacterial outer membrane porin	Key regulatory protein in two-component regulatory systems	0.005
LuxS	S-ribosylhomocysteine lyase	Involved in quorum sensing signal molecule synthesis	0.003
MntP	Manganese efflux pump	Mediates manganese efflux	0.011
KefG	Potassium efflux pump	Mediates potassium efflux	0.006
AhpC	Alkyl hydroperoxide reductase	Prevents cellular oxidative stress	0.001
SodA	Superoxide dismutase	Scavenges superoxides to protect cells from oxidative damage	0.005
IlvE	Branched-chain amino acid aminotransferase	Involved in protein synthesis and energy metabolism	0.006
AA320_08140	Deamido-glutathione amidase	Involved in glutathione metabolism and regulation	0.003
BfR	Bacterioferritin	Involved in iron ion utilization and management	0.021
PnuC	Nicotinamide nucleotide transporter	Maintains nicotinamide balance and plays important roles in metabolism	0.022
SitB	Iron/manganese ABC transporter ATP	Binding subunit–Facilitates transmembrane transport of metal ions such as iron and manganese	0.005

### 3.2 GO annotation and enrichment analysis

GO annotation and enrichment analysis were performed on all identified differentially expressed proteins (Figures 1C,D). The results showed that the biological processes predominantly involving these proteins were cellular processes and metabolic processes. These processes enable self-regulation and environmental adaptation by responding to external stresses such as oxidative stress, toxic substances, and pH changes. The main cellular components involved included cellular anatomical entities and intracellular structures. The primary molecular functions were concentrated in catalytic activity, binding, and transporter activity, which regulate nutrient supply and energy balance through the transport of nutrients and transmembrane transport systems. The GO annotation and enrichment analysis revealed the critical cellular activities, structural compositions, and molecular functions of the differentially expressed proteins during the formation of the SP (Planktonic) to BF (Biofilm) state.

### 3.3 KEGG pathway annotation and enrichment analysis

KEGG database analysis was employed to elucidate the biological pathways involving the differentially expressed proteins. The primary signaling pathways identified include (Figures 1C–E): “Cellular processes” (Biological processes—Quorum Sensing system, Biofilm formation); “Environmental information processing” (Signal transduction—Two-Component Systems, ABC transporter protein systems, bacterial secretion systems, etc.); “Genetic information processing” (Proteins involved in genetic information—Ribosome, Aminoacyl-tRNA biosynthesis, Transport proteins, etc.); “Human diseases” (Pathogenicity—Cationic antimicrobial peptide resistance, *Salmonella* infection, beta-Lactam resistance, etc.); “Metabolic pathways” (Substance metabolism—Metabolic pathways, Biosynthesis, Biosynthesis of secondary metabolites, Microbial metabolism in diverse environments, and Amino acid metabolism); and “Organismal systems” (Organismal systems—Longevity regulating pathway, Glucagon signaling pathway, PPAR signaling pathway, etc.). The KEGG annotation and enrichment analysis demonstrate that the differentially expressed proteins play significant roles in signal transduction, metabolic pathways, pathogenicity, and survival adaptation during the SP (Planktonic) to BF (Biofilm) transition.

### 3.4 COG annotation analysis

COG annotation was performed on the differentially abundant proteins, with the specific results shown in Figure 1F. The COG annotation revealed that the differentially abundant proteins were primarily concentrated in functional categories such as carbohydrate transport and metabolism, energy production and conversion, and amino acid transport and metabolism. This indicates that the formation of SP-BF involves active metabolic processes and nutrient metabolic pathways.

### 3.5 Validation of the differential proteomics results

**Figure 2:**
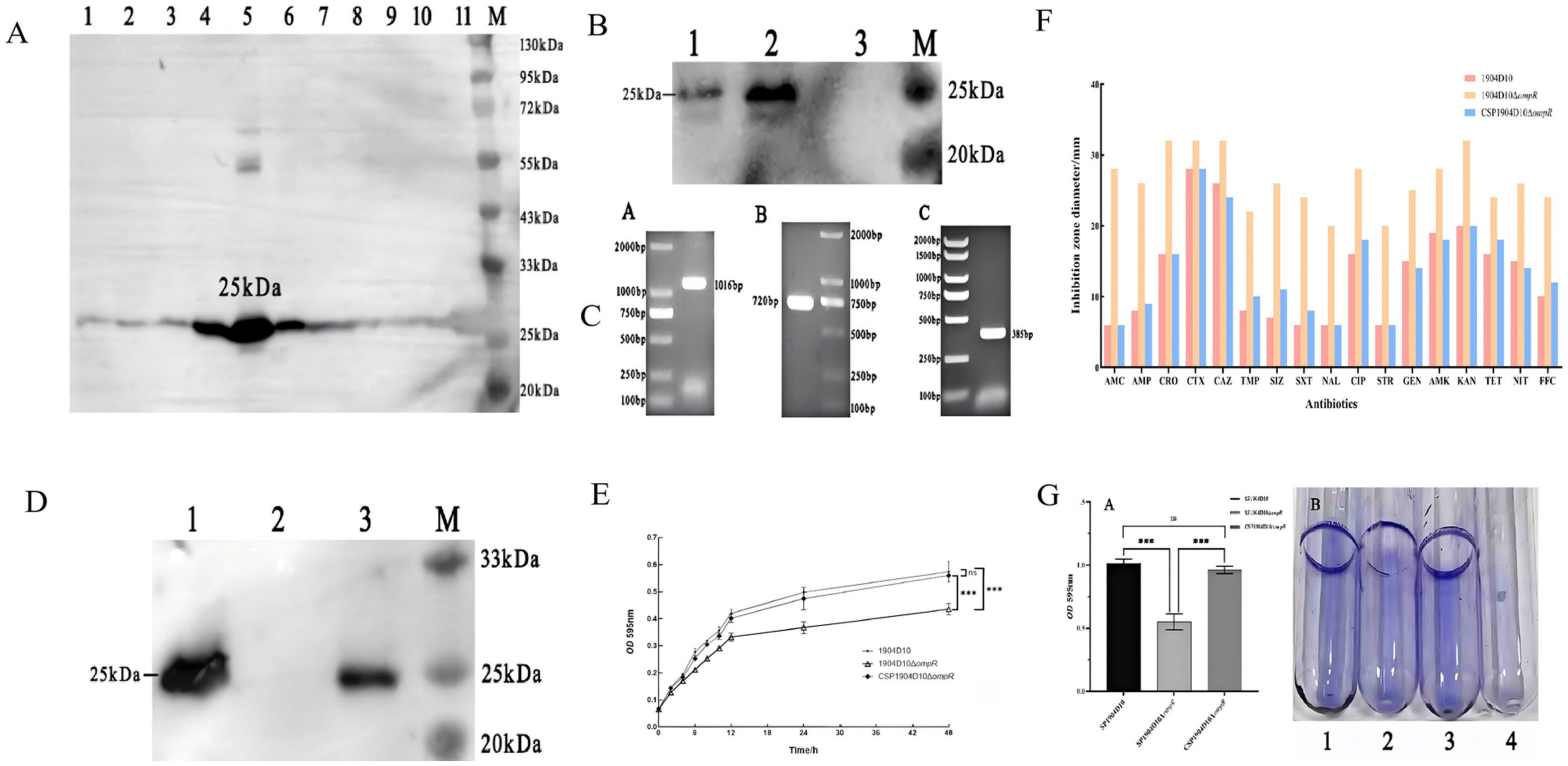
Regulation of SP-BF formation by the EnvZ/OmpR two-component system. **(A)** Purification of OmpR recombinant protein (1–6: Different native elution buffer with the concentrations of 20, 60, 100, 200, 250, 500 mmol/L, respectively; 7–10: Eluent; 11: Washing solution. M: Protein marker). **(B)** Differential expression levels of OmpR in planktonic and BF states of strain 1904D109(1: Planktonic state strain; 2: Biofilm state strain; 3: Blank control; M: Protein marker). **(C)** The identification of *ompR* gene mutant strain and complementation strains by PCR(A: pKD3-ompR; B: Complementary gene; C: CSP1904D10∆*ompR*). **(D)** WB identification of 1904D10 *ompR* gene mutant strain and complementation strains (1: SP1904D10; 2: SP1904D10 ∆*ompR*; 3: CSP1904D10 ∆*ompR*; M: Protein Marker). **(E)** Growth curve of 1904D10∆*ompR*. **(F)** Antibiotic resistance phenotype of 1904D10Δ*ompR* mutant strain. G: Impact of *ompR* Gene mutant on the BF formation ability of SP [**(B)** 1: SP1904D10; 2: SP1904D10Δ*omp*R; 3: CSP1904D10Δ*ompR*; 4: Blank control].

As shown in Figure 2A, the OmpR protein was successfully expressed and purified *in vitro*, presenting a relatively single target band at approximately 25 kDa. Furthermore, Western blot (WB) analysis confirmed the results of the differential proteomics study (Figure 2B), demonstrating that the expression level of the OmpR protein in the 1904D10 strain was higher in the biofilm (BF) state compared to the planktonic state. In addition to OmpR, proteomics analysis identified other differentially expressed proteins involved in quorum sensing (e.g., LuxS) and efflux pump systems (e.g., MntP, KefG, PnuC, SitB) (Table 2). Although independent validation of these proteins (e.g., by qPCR or Western blot) was not performed in this study, their consistent up-regulation in the biofilm state suggests the potential involvement of these systems in SP biofilm formation. Future studies are needed to validate these candidate proteins.

### 3.6 Construction of an *ompR* gene deletion mutant in SP isolate 1904D10

Figure 2C shows the PCR identification results of the 1904D10∆*ompR* mutant. The successful construction of the 1904D10∆*ompR* strain and its complementary strain, CSP1904D10∆*ompR*, was further verified by Western blot analysis (Figure 2D).

### 3.7 Deletion of the *ompR* gene affects the growth of SP strains

The growth curves of strains 1904D10, 1904D10∆*ompR*, and CSP1904D10∆*ompR* are shown in Figure 2E. While the growth rates of the parental strain and the complementary strain were similar, the growth rate of 1904D10∆*ompR* during the logarithmic phase was significantly lower than that of the parental and complementary strains. Furthermore, the overall trajectory of the growth curve for 1904D10∆*ompR* remained lower than that of both the parental and complementary strains.

### 3.8 Deletion of the *ompR* gene affects the antibiotic resistance phenotype of SP strains

As shown in Figure 2F, deletion of the *ompR* gene resulted in the parental strain 1904D10 becoming susceptible to antibiotics to which it was previously tolerant, such as AMC, AMP, TMP, SIZ, SXT, NAL, and STR. Furthermore, susceptibility to all other tested antibiotics was significantly increased following the loss of the *ompR* gene.

### 3.9 Deletion of the *ompR* gene affects the BF formation ability of SP strains

The results of the biofilm formation assay (Figure 2G) show that the parental strain and the complementary strain produced comparable amounts of biofilm, while deletion of the *ompR* gene in SP significantly reduced biofilm formation.

### 3.10 The EnvZ protein can bind to the AI-2 signaling molecule of the QS system

**Figure 3:**
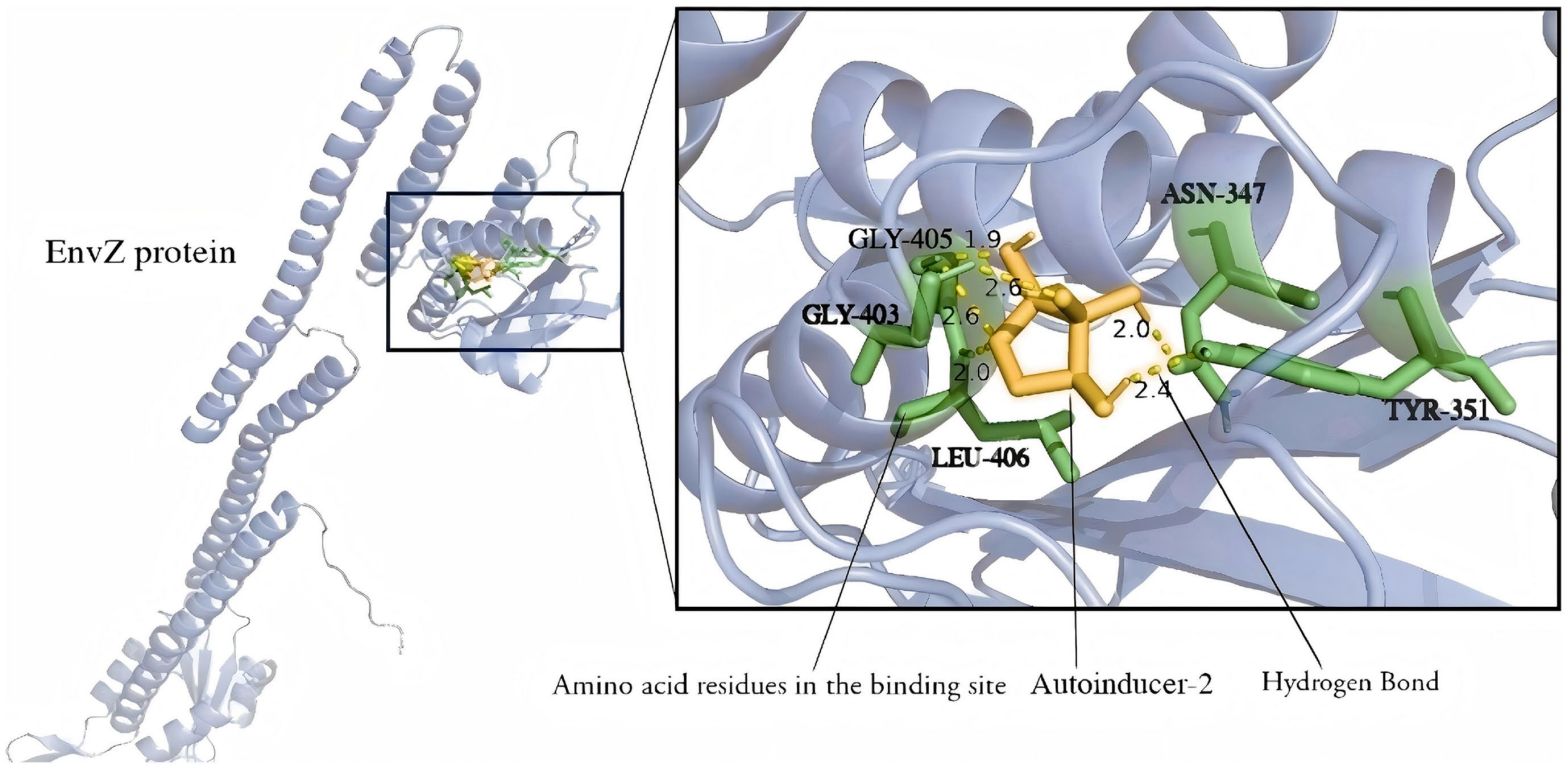
Analysis of interaction between AI-2 and EnvZ protein.

The binding energy between the EnvZ protein and the AI-2 signaling molecule was determined to be −23.99 kJ/mol using AutoDock Vina software, indicating significant binding affinity. Analysis of the binding site showed that the AI-2 molecule forms stable hydrogen bonds with five amino acid residues of the EnvZ protein. The molecular docking model clearly illustrates their interaction patterns (Figure 3). This suggests that AI-2 may promote EnvZ phosphorylation to enhance TCS activity, thereby promoting SP-BF formation.

### 3.11 The OmpR protein binds to the promoter region of the *soxR* efflux pump gene

**Figure 4:**
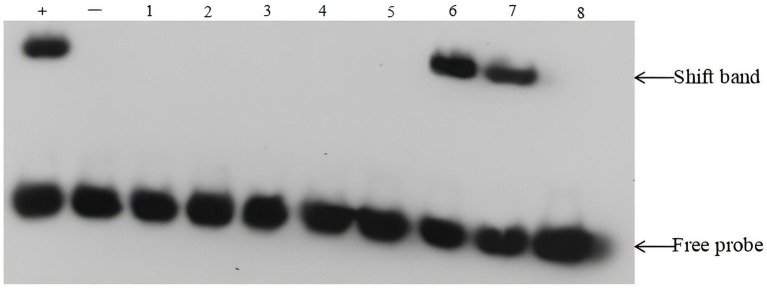
The binding of OmpR protein to the promoter of *soxR* gene.

The interaction results between the OmpR protein and the promoter regions of the efflux pump regulatory genes *soxS* and *soxR* are shown in Figure 4. While no binding was observed between the OmpR protein and the *soxS* gene promoter probe, a clear binding interaction was detected with the *soxR* gene promoter probe. Specifically, a 50-fold excess of the unlabeled wild-type *soxR* competitor probe partially competed for protein binding, and a 100-fold excess completely abolished the binding. These findings suggest that the OmpR protein likely regulates the expression of the *soxR* gene by binding to its promoter region but does not directly affect the transcription of the *soxS* gene.

## 4 Discussion

Pullorum disease is a critical infectious disease that urgently requires eradication in China’s breeding poultry industry. Statistical data indicate that the annual economic losses caused by pullorum disease far exceed those of other avian diseases, resulting in severe financial impacts on the poultry sector (Zhang et al., 2023). Studies have shown that approximately 80% of chronic infections in clinical settings are associated with the formation of BF. SP-BF can establish a cycle of contamination within farms. In particular, hatcheries and hatching rooms often concentrate large numbers of chicks. Chicks infected through vertical transmission continuously shed bacteria into the environment, while feathers, dust particles, and farming equipment provide surfaces for SP colonization, thereby establishing a persistent reservoir of BF. This leads to long-term horizontal transmission of SP within farms (Mueller-Doblies et al., 2013). Therefore, clarifying the biofilm-forming capacity and mechanisms of SP is essential for the eradication of pullorum disease in high-quality poultry breeds.

This study employs differential proteomics technology to gain in-depth insights into the biological functions, involved biological processes, key regulatory mechanisms, and protein–protein interactions of SP during BF formation. The aim is to further elucidate the underlying mechanisms of SP-BF development, thereby enhancing prevention and control strategies for pullorum disease. Simultaneously, by investigating the interactions among the EnvZ/OmpR TCS, the AcrAB-TolC efflux pump, and the LuxS/AI-2 QS system, this research seeks to reveal the cross-regulatory mechanisms involved in SP-BF formation. This will facilitate the identification of potential intervention targets at key stages of biofilm formation, offering novel perspectives and strategies to address persistent SP infections in clinical settings.

### 4.1 Differential proteomics analysis between planktonic and biofilm states of SP

To understand the global protein expression changes during SP biofilm formation, we performed TMT-based differential proteomics. Compared to planktonic bacteria and those in the superficial layer of the BF, bacteria in the deeper layers of the BF lack oxygen and sufficient nutrients, which triggers their stress response to resist environmental pressures and antibiotic stress. Under such extreme conditions, bacteria must adapt to environmental changes by regulating the expression of relevant proteins, thereby enhancing their own tolerance to stress. This study utilized differential proteomics to detect differentially expressed proteins in SP under planktonic and BF states, identifying a total of 219 differential proteins (Figure 1A). Among these, 96 proteins were upregulated, while 123 were down regulated. Table 2 lists a selection of proteins that were up-regulated in strain 1904D10 upon biofilm formation relative to its planktonic state. Analysis of the differential proteomics results showed that the expression levels of proteins related to a series of regulatory systems, oxidative stress, metabolic regulation, DNA damage repair, and transport functions were generally upregulated.

Biofilm formation is a complex biological process regulated by multiple factors within the bacterial internal and external environment. Within the bacterial population, signal transduction systems play a key role in biofilm formation. Among the differentially expressed proteins, key proteins involved in regulatory systems such as signal transduction systems, QS systems, and efflux pump systems tend to exhibit upregulation. For instance, the expression level of OmpF protein is increased under the regulation of the EnvZ/OmpR TCS, thereby modulating bacterial adaptation to osmotic stress. Similarly, Shimada et al. (2015) confirmed that the EnvZ/OmpR TCS influences bacterial biofilm formation by regulating the expression of multiple genes, thus playing a significant role as a signal transduction system during biofilm formation. Likewise, the LuxS/AI-2 type QS system, as a major communication system among bacteria, was implicated in this differential protein screening, with the identification of related proteins such as LuxS protease encoded by the luxS gene showing upregulated expression. This confirms the active involvement of the QS system in key physiological processes such as bacterial group behavior and biofilm formation. Proteomic studies on *Cronobacter* also revealed a significant upregulation of LuxS protein expression after biofilm formation (Ou et al., 2023). In addition to signal transduction systems, efflux pump systems also play an important role in bacterial survival strategies. Most bacteria require trace metal elements for growth. These nutrients are crucial for the catalytic activity of many key enzymes. However, excessive concentrations of metal elements can disrupt intracellular metal ion homeostasis, leading to toxic effects on cells (Heindl et al., 2016). Among the upregulated efflux pump system-related proteins in SP-BF, the manganese efflux pump protein MntP and the potassium efflux system regulatory protein KefG showed significantly increased expression under biofilm conditions, thereby maintaining normal cellular life activities. These differentially expressed proteins may participate in key pathways and regulatory networks involved in biofilm formation, playing important roles in bacterial adhesion, aggregation, and biofilm structure development. Studies have confirmed that mutations in proteins within TCS significantly reduce the biofilm-forming ability of bacterial strains. Moreover, inhibitors targeting key proteins in the QS system are currently a research focus, as they can serve as antibacterial synergists to reduce antibiotic use (Asif, 2020). Therefore, the aforementioned differentially expressed proteins may serve as potential targets for inhibiting biofilm formation.

In addition to signal transduction and efflux pump systems, metabolic regulation also plays a crucial role in bacterial biofilm formation. SP-BF adapt to adverse environmental factors through a series of physiological and metabolic adjustments. Differential proteomic data revealed the upregulation of oxidative stress-related proteins, such as alkyl hydroperoxide reductase subunit C (AhpC) and superoxide dismutase (SodA). When exposed to oxidative stress, proteins like AhpC and SodA may alleviate oxidative damage by scavenging intracellular reactive oxygen species, thereby promoting bacterial survival and growth under unfavorable conditions. Studies have shown that SodA plays a critical role in resisting environmental stress, epithelial cell adhesion, and invasion. SodA is also a key protein involved in biofilm formation, adhesion, and host invasiveness. Mutation of the *sodA* gene significantly reduced the biofilm-forming ability of *S. typhimurium* (Yang et al., 2018). Furthermore, among the differentially expressed proteins identified in this study, metabolism-related proteins such as IlvE (involved in sulfur-amino acid metabolism), the hydrolytic enzyme AA320_08140 (important in detoxification metabolism), and the essential iron storage protein Bfr (involved in iron metabolism) were significantly upregulated (Rivera, 2017). These proteins may enhance bacterial metabolic activity, thereby regulating bacterial growth and biofilm formation. Notably, a series of transport proteins, such as the nicotinamide mononucleotide transporter PnuC and the ATP-binding protein SitB of the iron/manganese ABC transporter system, also exhibited increased expression. These upregulated transport proteins may provide the bacteria with enhanced access to iron and other essential nutrients. In summary, the formation of SP-BF involves alterations in the expression levels of a range of proteins related to oxidative stress, metabolism, and transport, providing important insights into the molecular mechanisms underlying SP-BF formation.

This study reveals that the differentially expressed proteins in SP following biofilm BF formation encompass major proteins within key regulatory systems, including signal transduction, QS systems, and efflux pumps, further highlighting the critical role of these regulatory networks during BF development. Additionally, the significant upregulation of oxidative stress-related proteins, as well as proteins involved in metabolism and transport, demonstrates the physiological adaptation and metabolic remodeling that occur during bacterial BF formation. While differentially expressed proteins post-BF formation have been identified, the specific regulatory mechanisms and functional contributions of these proteins to the BF formation process remain poorly understood. Therefore, further investigation is essential to advance our understanding of the molecular mechanisms underlying BF formation.

### 4.2 Regulatory role of EnvZ/OmpR TCS in SP-BF formation

To further investigate the function of EnvZ/OmpR TCS in SP biofilm formation, we constructed an ompR deletion mutant. Adaptive regulation is critical for bacterial survival under changing environmental conditions. The EnvZ/OmpR TCS has been a key focus in studying bacterial perception and response to environmental shifts, as first demonstrated by Forst et al. (1989). As a crucial signal transduction system, it plays a central role in regulating bacterial BF formation. The findings above demonstrate that the EnvZ/OmpR TCS enhances BF formation in SP by modulating the expression of the *ompF* gene. Moreover, preliminary screening for target genes in SP isolates revealed that *ompR* is a conserved gene in SP. Previous studies by Lu et al. (2012) and Aleksandrowicz et al. (2023) confirmed that deletion of the *ompR* gene completely abolishes BF formation due to the inability to synthesize pili and cellulose. Therefore, the functional mechanism of the *ompR* gene in bacterial adaptive regulation and its impact on BF-forming capacity warrant further in-depth investigation.

Red homologous recombination is a widely used gene-editing technique employed to achieve targeted gene deletions in bacteria and other microorganisms. By introducing a recombinase system, this method enables bacteria to utilize exogenous DNA fragments to undergo recombination with homologous sequences in the target genome, thereby facilitating the deletion or replacement of specific genes. In this chapter, the *ompR* gene deletion mutant of SP was successfully constructed via Red homologous recombination. Analysis of the biological characteristics of the deletion mutant revealed that the growth rate of the *ompR* mutant during the logarithmic phase was significantly lower than that of the parental strain and the complementary strain (Figure 2E). This indicates that the absence of OmpR, a key transcription factor, disrupts the expression of genes associated with bacterial growth, thereby impairing the strain’s growth capacity. These findings are consistent with results reported by Kong et al. (2018). Additionally, the deletion of the *ompR* gene enhanced the susceptibility of SP to antibiotics, likely due to a diminished ability to perceive and respond to environmental changes. Similarly, Park et al. (2023) observed increased tigecycline sensitivity in an *ompR* deletion mutant of *Klebsiella pneumoniae* (Figure 2F). This may be attributed to increased outer membrane permeability resulting from the loss of OmpR, thereby elevating bacterial sensitivity to antibiotics. Furthermore, the absence of the *ompR* gene may also impair nutrient uptake and metabolic pathways, subsequently reducing BF formation. Relevant studies have demonstrated that deletion of ompR significantly attenuates BF formation in *Yersinia enterocolitica*. In summary, deletion of the *ompR* gene likely compromises the growth performance, antibiotic resistance, and BF-forming capacity of SP by interfering with signal transduction, increasing outer membrane permeability, and disrupting bacterial nutrient metabolism.

### 4.3 Predicted crosstalk between LuxS/AI-2 QS and EnvZ/OmpR TCS

To explore potential interactions between QS and TCS systems, we performed molecular docking between AI-2 and EnvZ. Additionally, we investigated the regulatory cascade involving EnvZ/OmpR TCS and the AcrAB-TolC efflux pump. The formation of SP-BF is regulated by a variety of factors, including environmental conditions, signaling molecules, and regulatory proteins, necessitating a comprehensive consideration of the complex interactions among these regulatory systems. Therefore, this study employed AutoDock software to investigate the potential relationship between the LuxS/AI-2-type QS system and the EnvZ/OmpR TCS in bacteria. The results indicate that the AI-2 signaling molecule of the QS system binds to the EnvZ protein of the TCS via hydrogen bonding, demonstrating a strong interaction between the two. This finding suggests that the AI-2 signaling molecule may promote the phosphorylation of EnvZ through binding, thereby participating in the bacterial response to environmental changes via the TCS. It has been confirmed that Gram-negative bacteria can establish a signal regulatory cascade through the interplay between the TCS and QS systems, jointly influencing biofilm formation. Although AI-2 lacks the ability to directly regulate gene transcription, exogenous supplementation of AI-2 has been shown to enhance bacterial growth rate and biofilm formation capacity, indicating that AI-2 may function through alternative pathways during bacterial biofilm development (Liu, 2018). The results of this study further support this perspective.

Efflux pump systems influence the process of bacterial BF formation by promoting the expression of polysaccharide proteins or the efflux of QS system signaling molecules, thereby accelerating the production of extracellular polymeric substances. Alternatively, they may directly upregulate the expression of genes associated with BF formation to exert their effects. Moreover, efflux pump systems are also subject to regulation by TCS. For instance, the AdeS/AdeR TCS enhances bacterial drug resistance by modulating the expression of the RND efflux pump gene *adeABC* (Liang et al., 2023). In this context, the present study preliminarily investigated the regulatory role of the EnvZ/OmpR TCS on the AcrAB-TolC efflux pump system in SP. EMSA results demonstrated that OmpR, a transcription factor of the TCS, can bind to the promoter region of the soxR gene, leading to the upregulation of SoxR protein expression (Figure 4). As a transcriptional activator, SoxR promotes the activation of the AcrAB-TolC efflux pump system under oxidative stress conditions, thereby enhancing the BF formation capacity of SP. These findings align with the report by Wang et al. (2022), which confirmed that the response regulator FlmD similarly promotes BF formation in *Comamonas testosteroni* through the transcriptional activator SoxR. The results of this study further elucidate the regulatory cascade involving the EnvZ/OmpR TCS and the AcrAB-TolC efflux pump system in the process of SP-BF formation.

The results of this study demonstrate that the EnvZ/OmpR TCS plays a critical role in the BF formation of SP. Deletion of the *ompR* gene impaired bacterial growth performance, increased susceptibility to antimicrobial agents, and attenuated BF-forming capacity. Furthermore, this study revealed a strong interaction between the AI-2 signaling molecule of the LuxS/AI-2-type QS system and the EnvZ protein of the EnvZ/OmpR TCS. This suggests that AI-2 may enhance TCS activity by promoting the phosphorylation of EnvZ, thereby facilitating BF formation. Additionally, this study preliminarily verified that the OmpR protein of the EnvZ/OmpR TCS system activates the AcrAB-TolC efflux pump system by upregulating the expression of the transcriptional activator SoxR. These findings further enrich our understanding of the regulatory mechanisms involving TCS, QS systems, and efflux pump systems in bacterial BF formation. Through pathways such as enhancing intercellular signaling and promoting gene expression, these systems collectively provide bacterial protection, promote SP-BF formation, and enhance the competitive advantage of SP in complex environments such as poultry farms.

Based on these findings, we propose a working model: Environmental signals → EnvZ senses → OmpR phosphorylated → OmpR binds soxR promoter → SoxR upregulates AcrAB-TolC → enhanced biofilm formation and antibiotic resistance. Concurrently, AI-2 quorum sensing molecules may interact with EnvZ, potentially modulating TCS activity.

### 4.4 Study limitations

While our proteomics analysis identified multiple differentially expressed proteins involved in quorum sensing and efflux pump systems, experimental validation in this study was limited to OmpR. The proposed roles of LuxS and efflux pump-related proteins in SP biofilm formation, though supported by proteomics data, require independent confirmation (e.g., by qPCR or Western blot) in future studies. Additionally, the predicted interaction between AI-2 and EnvZ is based solely on molecular docking and awaits functional validation. Future studies should also validate other candidate proteins identified in this study and investigate the detailed molecular mechanisms of the proposed regulatory network.

## 5 Conclusion

In summary, regulatory systems such as the TCS, QS system, and efflux pumps collectively modulate the formation of SP-BF. The EnvZ/OmpR TCS contributes to biofilm development and enhances antibiotic resistance by regulating the expression of porin proteins. Meanwhile, molecular docking suggests that the AI-2 signaling molecule of the QS system may interacts with the EnvZ protein of the TCS, promoting influencing its phosphorylation and thereby amplifying the regulatory effect of the TCS on downstream genes. Furthermore, the OmpR protein binds to the promoter of the *soxR* gene, upregulating the expression of the SoxR transcriptional activator, which in turn activates the AcrAB-TolC efflux pump system. These coordinated mechanisms synergistically facilitate SP-BF formation. This study provides critical insights into the molecular mechanisms underlying SP-BF formation, offering a scientific basis for developing more effective breeding management strategies and prevention and control measures against SP-BF in clinical and agricultural settings.

## Data Availability

The raw data supporting the conclusions of this article will be made available by the authors, without undue reservation.

## Footnotes

1
https://www.uniprot.org/

## Glossary

AMR: antimicrobial resistance
SP: *Salmonella pullorum*
QS: quorum sensing
PD: Pullorum disease
AI-2: autoinducer-2
AMC: amoxicillin
AMP: ampicillin
CRO: ceftriaxone
CTX: cefotaxime
CAZ: ceftazidime
TMP: methoxypyrimidine
SIZ: sulfaisoxazole
SXT: trimethoprim/sulfamethoxazole
NAL: nalidixic acid
CIP: ciprofloxacin
STR: streptomycin
AMK: amikacin
KAN: kanamycin
TET: tetracycline
NIT: nitrofurantoin
FFC: florfenicol
GEN: gentamicin
qPCR: quantitative polymerase chain reaction
PBS: phosphate-buffered saline
EMSA: electrophoretic mobility shift assay.

## References

[ref1] Aleksandrowicz A. Carolak E. Dutkiewicz A. Waszczuk A. Grzymajlo K. (2023). Better together–Salmonella biofilm-associated antibiotic resistance. Gut Microbes 15:2229937. doi: 10.1080/19490976.2023.2229937, 37401756 PMC10321201

[ref2] Asif M. (2020). Natural anti-quorum sensing agents against Pseudomonas aeruginosa. Chem. Rev. 2, 57–69. doi: 10.33945/sami/jcr.2020.1.4, 42154458

[ref4] Forst S. Delgado J. Inouye M. (1989). Phosphorylation of OmpR by the osmosensor EnvZ modulates expression of the ompF and ompC genes in *Escherichia coli*. Proc. Natl. Acad. Sci. USA 86, 6052–6056. doi: 10.1073/pnas.86.16.6052, 2668953 PMC297773

[ref6] Heindl J. E. Hibbing M. E. Xu J. Natarajan R. Buechlein A. M. Fuqua C. (2016). Discrete responses to limitation for iron and manganese in Agrobacterium tumefaciens: influence on attachment and biofilm formation. J. Bacteriol. 198, 816–829. doi: 10.1128/JB.00668-15, 26712936 PMC4810603

[ref7] Huang P. Zhu J. Li H. Wan Y. Z. Tang Y. M. Liu Q. (2022). Bioinformatic analysis of differentially expressed proteins in the dorsal raphe nucleus ofrats after continuous treatment with olanzapine. J. South. Med. Univ. 42, 1221–1229. doi: 10.12122/j.issn.1673-4254.2022.08.15, 36073222 PMC9458534

[ref12] Kong H. K. Pan Q. Lo W. U. Liu X. Law C. O. K. Chan T. F. . (2018). Fine-tuning carbapenem resistance by reducing porin permeability of bacteria activated in the selection process of conjugation. Sci. Rep. 8:15248. doi: 10.1038/s41598-018-33568-8, 30323356 PMC6189183

[ref13] Liang J. Z. (2019). Detection of Salmonella in Breeder Companies and Experimental Study on SYF Prevention and Control of Salmonella Infection. Master's thesis. Nanning: Guangxi University.

[ref14] Liang Z. B. Lin Q. Q. Wang Q. W. Huang L. H. Liu H. D. Shi Z. R. . (2023). Gram-negative bacteria resist antimicrobial agents by a DzrR-mediated envelope stress response. BMC Biol. 21, 62–76. doi: 10.1186/s12915-023-01565-7, 36978084 PMC10052836

[ref15] Liu Z. C. (2018). Regulatory Mechanisms of AI-2 quorum Sensing on Chlortetracycline Resistance in avian Pathogenic *Escherichia coli*. Master's thesis. Hefei: Anhui Agricultural University.

[ref16] Liu Y. H. (2021). Mechanisms of Endosymbiotic bacteria Regulating the Interaction between Phenacoccus solenopsis and cotton. Master's thesis. Wuhan: Huazhong Agricultural University.

[ref17] Lu Y. Chen S. J. Dong H. Y. Sun H. L. Peng D. X. Liu X. F. (2012). Identification of genes responsible for biofilm formation or virulence in *Salmonella enterica* serovar pullorum. Avian Dis. 56, 134–143. doi: 10.1637/10008-980611-DIGEST.1 22545539

[ref19] Mueller-Doblies D. Clouting C. Davie R. H. (2013). Investigations of the distribution and persistence of Salmonella and ciprofloxacin-resistant Escherichia coli in Turkey hatcheries in the UK. Zoonoses Public Health 60, 296–303. doi: 10.1111/j.1863-2378.2012.01524.x, 22856515

[ref20] Ou D. X. Ling N. Tong L. W. Zhang D. F. Shen Y. Ye Y. W. (2023). Comparative proteomic and phenotypic changes of LuxS-mediated hyperosmotic tolerance in *Cronobacter malonaticus*. Int. J. Dairy Technol. 76, 927–938. doi: 10.1111/1471-0307.12990

[ref22] Pandey A. Mann M. (2000). Proteomics to study genes and genomes. Nature 405, 837–846. doi: 10.1038/35015709, 10866210

[ref23] Park S. Kim H. Ko K. S. (2023). Reduced virulence in tigecycline-resistant Klebsiella pneumoniae caused by overexpression of ompR and down-regulation of ompK35. J. Biomed. Sci. 30, 22–33. doi: 10.1186/S12929-023-00910-W 37004036 PMC10064660

[ref24] Qiao W. B. (2020). Application of Slightly acidic Electrolyzed water in Peanut Sprouts. Master's thesis. Shijiazhuang: Hebei University of Science and Technology.

[ref26] Rivera M. (2017). Bacterioferritin: structure, dynamics, and protein–protein interactions at play in iron storage and mobilization. Acc. Chem. Res. 50, 331–340. doi: 10.1021/acs.accounts.6b00514, 28177216 PMC5358871

[ref27] Shimada T. Takada H. Yamamoto K. Ishihama A. (2015). Expanded roles of two-component response regulator OmpR *in Escherichia coli*: genomic SELEX search for novel regulation targets. Genes Cells 20, 915–931. doi: 10.1111/gtc.12282, 26332955

[ref28] Stepanović S. Vuković D. Dakić I. Savić B. Švabić-Vlahović M. (2000). A modified microtiter-plate test for quantification of staphylococcal biofilm formation. J. Microbiol. Methods 40, 175–179. doi: 10.1016/S0167-7012(00)00122-6, 10699673

[ref29] Wang C. (2022). Investigation of Salmonella Contamination in Guangxi high-Quality chicken Breeding Farms and Research on Antibiotic-Alternative Products. Master's thesis. Nanning: Guangxi University.

[ref30] Wang Y. H. Huang Z. Zhou N. Liu C. Jiang C. Y. Li D. F. . (2022). The response regulator FlmD regulates biofilm formation in Comamonas testosteroni through the transcriptional activator SoxR. Microorganisms 10, 356–372. doi: 10.3390/microorganisms10020356 35208812 PMC8880074

[ref31] Xu Z. H. (2022). Multidrug Resistance Mechanism of Salmonella Pullorum Based on LuxS/AI-2 Quorum Sensing System and the Antibacterial Sensitization Effect of QS Inhibitor Glycyrrhizic Acid. PhD Dissertation. Nanning: Guangxi University.

[ref32] Xu Z. Liang Y. Lin S. Chen D. Li B. Li L. . (2016). Crystal violet and XTT assays on *Staphylococcus aureus* biofilm quantification. Curr. Microbiol. 73, 474–482. doi: 10.1007/s00284-016-1081-1, 27324342

[ref33] Xu Z. H. Wang M. Zhou C. Y. Gu G. M. Liang J. Z. Hou X. J. . (2020). Prevalence and antimicrobial resistance of retail-meat-borne Salmonella in southern China during the years 2009–2016: the diversity of contamination and the resistance evolution of multidrug-resistant isolates. Int. J. Food Microbiol. 333:108790. doi: 10.1016/j.ijfoodmicro.2020.108790, 32693316

[ref34] Yang W. Li Y. Zhang J. K. Sun L. Y. Wen W. Y. Zhang C. J. . (2018). Functional analysis of superoxide dismutase of Salmonella typhimurium in serum resistance and biofilm formation. J. Appl. Microbiol. 125, 1526–1533. doi: 10.1111/jam.14044, 29989280

[ref35] Zhang G. S. Yang L. Zhang Y. Yang J. (2023). Transmission routes and prevention measures of avian pullorum disease. Mod. Rural Sci. Technol. 6, 67–67. doi: 10.3969/j.issn.1674-5329.2023.06.049

